# Weak Antilocalization Tailor-Made by System Topography in Large Scale Bismuth Antidot Arrays

**DOI:** 10.3390/ma13153246

**Published:** 2020-07-22

**Authors:** Michal Krupinski, Arkadiusz Zarzycki, Yevhen Zabila, Marta Marszałek

**Affiliations:** Institute of Nuclear Physics Polish Academy of Sciences, Radzikowskiego 152, 31342 Krakow, Poland; arkadiusz.zarzycki@ifj.edu.pl (A.Z.); Yevhen.Zabila@ifj.edu.pl (Y.Z.); Marta.Marszalek@ifj.edu.pl (M.M.)

**Keywords:** antidots, magnetotransport, weak antilocalization, magnetoresistance, Hall effect, semimetals

## Abstract

Using a two-carriers model and the Hikami-Larkin-Nagaoka (HLN) theory, we investigate the influence of large area patterning on magnetotransport properties in bismuth thin films with a thickness of 50 nm. The patterned systems have been produced by means of nanospheres lithography complemented by RF-plasma etching leading to highly ordered antidot arrays with the hexagonal symmetry and a variable antidot size. Simultaneous measurements of transverse and longitudinal magnetoresistance in a broad temperature range provided comprehensive data on transport properties and enabled us to extract the values of charge carrier densities and mobilities. Weak antilocalization signatures observed at low temperatures provided information on spin-orbit scattering length ranging from 20 to 30 nm, elastic scattering length of approx. 60 nm, and strong dependence on temperature phase coherence length. We show that in the absence of antidots the charge carrier transport follow 2-dimensional behavior and the dimensionality for phase-coherent processes changes from two to three dimensions at temperature higher than 10 K. For the antidot arrays, however, a decrease of the power law dephasing exponent is observed which is a sign of the 1D-2D crossover caused by the geometry of the system. This results in changes of scattering events probability and phase coherence lengths depending on the antidot diameters, which opens up opportunity to tailor the magnetotransport characteristics.

## 1. Introduction

Bismuth is a semimetal which has attracted substantial attention due to its distinctive electronic properties. These include low carrier density (few orders of magnitude smaller than those of most metals), small carrier effective mass of ~0.001 m_e_, high carrier mobility reaching ~10^3^ m^2^/Vs in pure crystals, and long carrier mean free path exceeding 1 μm in single crystals [1,2,3,4]. It is also an exceptional material for studying quantum mechanics in the solid state; many important physics phenomena, such as de Haas-van Alphen effect and quantum linear magnetoresistance, were first observed in Bi [5,6,7]. Due to a long quantum phase coherence length, and strong spin-orbit interaction Bi films serve as a model material in the area of condensed matter physics showing various interesting magnetotransport properties, such as weak antilocalization (WAL) [5,8,9,10,11] and large magnetoresistance (MR) up to 380,000% at 5 K and 5 T [12].

These physical properties are used for applications in electronics and give rise to a number of studies devoted to the fabrication of low dimensional Bi structures and their magnetotransport properties [11,13]. In particular, one-dimensional nanowires attracted a lot of attention [2,4,14,15,16,17,18], demonstrating the broad potential for fundamental investigations as well as a variety of applications such as next-generation quantum computing devices [19,20]. Another interesting system in this context are the arrays of Bi antidots, in which the correlation between the dimensionality and electronic properties was observed [3]. However, the studies of such systems are sparse and almost exclusively related to Bi films deposited on anodic aluminum oxide (AAO) matrices, which are known for their developed topography and small ordered domains with sizes of only a few micrometers [3,21,22]. Alternatively, the hole arrays made by optical lithography have been investigated, but this approach does not allow to obtain antidots with diameter smaller than 1 μm [23]. The difficulties in fabrication of the high quality large area arrays of Bi antidots with submicron periods make such studies demanding and are one of the obstacles to the use of such systems in electronics and optoelectonics. In the latter case developing of optically transparent magnetic sensors is an important challenge. One of the considered solutions is to create a regular nano-perforation network (antidots) in thin films of materials, such as bismuth. This approach offers the combination of good magnetic properties with flexibility and tunable light transmittance, which is desirable for integrated hybrid opto-magnetic sensors with various functionalities [24,25].

In this study, we focused on the Bi antidot arrays fabricated by nanosphere lithography (NSL). This approach provides hexagonally-ordered arrays with areas larger than a few cm^2^, and offers high scalability at low cost. The diameter of antidots was altered with support of the RF-plasma etching, which allowed us to adjust the dimensionality of the system and its transport parameters. The use of a double cross mask during the deposition of the films enabled simultaneous measurement of both the transverse and the longitudinal magnetoresistance, which provided comprehensive data on the transport properties. Additionally, the inclusion of mesoscopic defects serving as scattering centers enabled the observation of WAL effect tuned by the size of antidots. The electrical properties of the arrays were then compared to those of the continuous films in order to determine the impact of patterning on magnetotransport properties.

## 2. Materials and Methods

Hexagonally ordered arrays of Bi antidots were fabricated using a NSL technique. The detailed description of the process can be found in the previous publications [26,27]. Briefly, polystyrene (PS) nanospheres with a diameter of 175 nm were applied to the surface of water, where a close-packed monolayer was formed by self-assembly. Then, the monolayer was transferred onto the Si/SiO_2_ (100 nm) substrate and dried in air. In order to decrease the diameter of the PS spheres, RF-plasma etching (MiniFlecto, Plasma Technology GmbH, Herrenberg, Germany) was used. The plasma process was performed in oxygen and argon atmosphere at the pressure of 0.15 mbar. Three different durations of etching of 3, 4, and 5 min were applied resulting in a mean particle size of 110, 65 and 45 nm, respectively.

**Figure 1:**
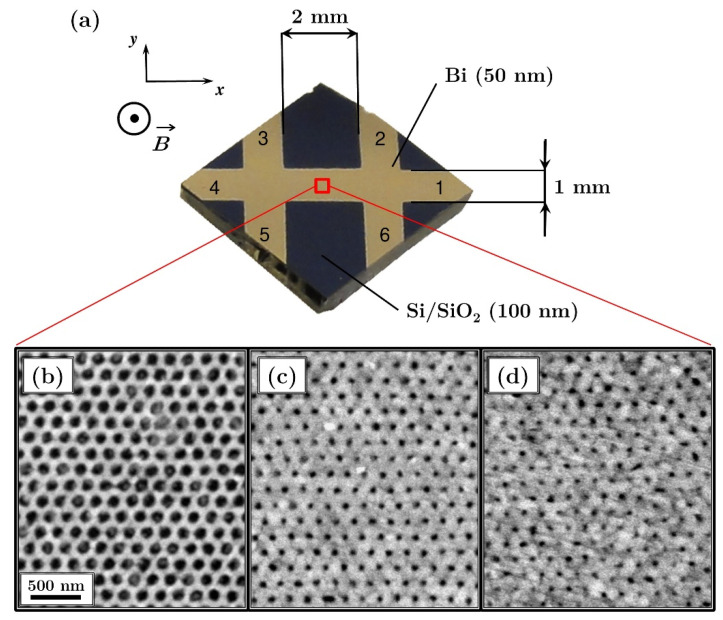
(**a**) Photograph of a sample showing dimensions and geometry of the double cross Hall bar. Below, scanning electron microscopy (SEM) image of a Bi antidot array with a period of 175 nm. The measured mean hole diameter is (**b**) 110 nm, (**c**) 65 nm, and (**d**) 45 nm.

Next, a 50-nm-thick Bi film was deposited by thermal evaporation onto the size-reduced PS nanospheres serving as a shadow mask. The value of evaporation rate of 5 Å/s was adopted, while the working pressure was at the level of magnitude of 10^−6^ mbar. The film thickness was controlled in situ by a quartz microbalance and ex situ by x-ray reflectometry. In addition to the patterned samples, 50 nm Bi film was also deposited onto a flat Si/SiO_2_ (100 nm) substrate for reference. The parameters of the deposition were the same as described above. In all cases, the deposition was carried out through a double-cross mask placed directly on the substrate, and was followed by removal of the nanospheres using ultrasonic assisted lift-off. This led to the formation of the large area well-ordered Bi antidot arrays with six contacts ready for magnetotransport measurements, as shown in Figure 1a. The scanning electron microscopy (SEM) (Vega3 SB, Tescan, Brno Czech Republic) images presented in Figure 1b–d indicate high order of the obtained arrays of antidots with various diameters from 45 nm to 110 nm. To reduce the oxidation, the samples were stored in a desiccator and magnetotransport measurements were carried out within two days of the deposition. This approach provides the oxide layer thickness of less than 1 nm, which was confirmed by the X-ray reflectivity measurements (not shown).

Four-point magnetoresistance and Hall measurements were conducted with a commercial SQUID magnetometer equipped with a manual insertion utility probe provided by Quantum Design (MPMS XL, San Diego, CA, USA). In all cases the external magnetic field was applied perpendicular to the sample surface and varied in the range of ±70 kOe, while the temperature range was 2 K < *T* < 300 K. The use of a double cross enabled simultaneous measurement of longitudinal magnetoresistance and of the Hall effect. The measurements were performed with the current of 0.1 mA applied between contacts 1 and 4 (see Figure 1a). The voltages between contacts 2–3 and 6–5 were used to determine longitudinal magnetoresistivity *ρ_xx_*(*B*), while the voltages between contacts 3–5 and 2–6 were used to obtain values of transverse magnetoresistivity *ρ_xy_*(*B*) and the magnitude of Hall effect.

## 3. Results and Discussion

### 3.1. Non-Patterned Film

The resistance of the flat reference layer measured at 300 K at zero magnetic field was 337 Ω, which gives the resistivity of 8.4 × 10^−6^ Ωm, more than for a single crystal bulk Bi (1.1–1.3 × 10^−6^ Ωm) [28,29,30]. One can expect it for the polycrystalline thin films (see Figure S1 in Supplementary Materials), since the resistivity value is influenced by grain-boundary scattering and depends on the grain sizes. Similar values of resistivity for Bi layers grown by thermal evaporation on the Si/SiO_2_ substrates were observed by Marcano et al. [31].

**Figure 2:**
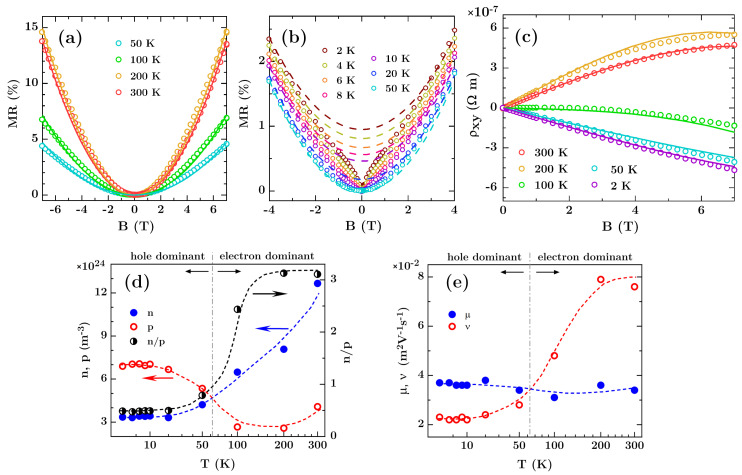
(**a**,**b**) Longitudinal magnetoresistance (MR) vs field curves and (**c**) Hall transverse resistivity dependences for the non-patterned Bi film with thickness of 50 nm. Solid and dashed lines represent theoretical curves fitted to experimental data using the two-carriers model. For clarity, the results of Hall measurements for temperature range 4 K–20 K were omitted since they are similar to those obtained at 2 K and 50 K. (**d**,**e**) Densities and mobilities of electrons and holes derived from the fitting. The right scale of graph (**d**) is related to the n/p ratio.

The magnetoresistance (MR) and transverse (Hall) component of the resistivity measured in the range of −7 T < *B* < 7 T for various temperatures are depicted in Figure 2a–c. Figure 2a shows the magnetoresistance results for temperatures 50 K and higher, while Figure 2b depicts the magnetoresistance at a low temperature range from 2 to 50 K. MR is defined as MR(%) = 100 × [*ρ_xx_*(*B*,*T*) − *ρ_xx_*(*B* = 0,*T*)]/*ρ_xx_*(*B* = 0,*T*) and shows quadratic behavior without saturation for temperatures above 10 K, which is a typical attribute seen in the classical MR phenomena. Deviations from the classical behavior are only observed for the low magnetic field below 2 T at temperatures below 20 K. The largest MR of up to 15% appears at 200 K, which is three times higher than that at 2 K and is comparable to values reported in other works [9,10,21,22].

The Hall curves in entire temperature range are symmetrical with respect to the origin under negative and positive magnetic field, so only one half of the characteristics is presented (see Figure 2c). The evolution of the Hall curves with temperature and change of the sign of Hall coefficient clearly show a transition from p-type to n-type conduction with the increasing temperature. Such behavior can be described in the framework of the two-carrier model, where the longitudinal and transverse components of the resistivity are given as [32,33,34]:

(1) $$\rho_{xx}=\frac{1}{e}\frac{\left(p\nu +n\mu \right)+\left(n\mu \nu^{2}+p\nu \mu^{2}\right)B^{2}}{{\left(p\nu +n\mu \right)}^{2}+\mu^{2}\nu^{2}B^{2}{\left(p-n\right)}^{2}}$$

(2) $$\rho_{xy}=\frac{1}{e}\frac{\left(p\nu^{2}-n\mu^{2}\right)+\mu^{2}\nu^{2}B^{2}\left(p-n\right)}{{\left(p\nu +n\mu \right)}^{2}+\mu^{2}\nu^{2}B^{2}{\left(p-n\right)}^{2}}B$$

where *n* (*p*) and *μ* (*ν*) are the density and mobility of electrons (holes), respectively.

In order to extract the carrier parameters *n*, *p*, *μ*, and *ν*, the measured magnetoresistance data and Hall resistance curves were simultaneously fitted to Equations (1) and (2). The solid and dashed lines in Figure 2a–c represent theoretical curves of *ρ_xy_*(*B*), and *ρ_xx_*(*B*) converted to MR, fitted in the range of −7 T < *B* < 7 T, while the extracted carrier densities and mobilities are displayed in Figure 2d,e. The theoretical curves successfully reproduce both the B-quadratic increase of MR(*B*) and *ρ_xy_*(*B*) dependencies for values of *n* and *p* of the order of 10^24^ m^−3^. They correspond to numbers of electrons and holes in bismuth single crystals and in the pure Bi films, which is known to be in the range of *n* ≈ *p* ∼ 10^23^–10^24^ m^−3^ [35,36]. At 300 K the majority carrier are electrons but holes have bigger mobility, which corroborates earlier findings [34,36,37,38]. Similar electron and hole mobilities as estimated by us, were also found for Bi samples prepared in other conditions and by different deposition methods [34,36,37].

The electron to holes density ratio *n*/*p* depends on temperature and changes from the value of approximately 0.5 at *T* < 20 K in the hole dominant regime to the value of 3 at room temperature in the electron dominant regime. The temperature of transition from p-type of conductivity to n-type of conductivity was between 50 K and 100 K and similar behavior was observed for 300-nm-thick polycrystalline bismuth films [31]. As shown in Figure 2e the transition is accompanied by the changes in the electron and hole mobilities, corresponding to those reported by Dillner et al. [35]. The obtained mobility values for temperatures below 10 K correlate well also with the results obtained in [32] for the ultrathin bismuth layers. However, it should be noted that our results are only an estimation, since a full description of the bismuth thin film conductivity can be obtained only by the use of a three-band model with electron and hole bulk bands accompanied by a surface electron band [37]. Such surface carriers, however, are expected to dominate the electronic transport only in ultrathin Bi films with thickness of 10 nm and lower [32,37]. For this reason, in the case which we discuss here the two-carrier analysis can be successfully used for describing the field-dependent resistivity. As shown by Marcano et al. [37] for the thicker layers these two models do not differ much, and the two-carrier model should reproduce well the magnetotransport of bismuth layers with thickness of 50 nm and around.

**Figure 3:**
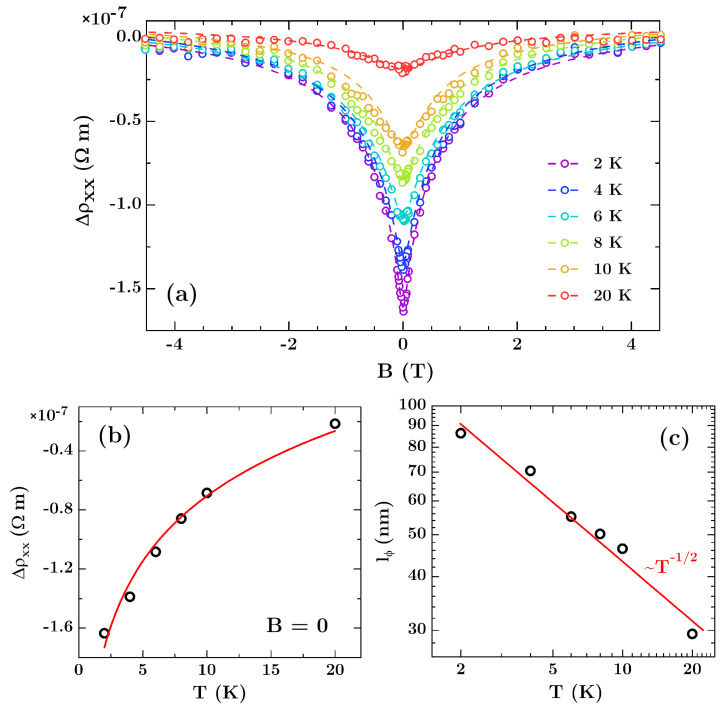
(**a**) Longitudinal magnetoresistivity for the non-patterned Bi film of 50 nm after subtracting the B-quadratic background. Dashed lines represent theoretical curves fitted to experimental data using Equation (4) and HLN theory. (**b**) Resistivity deviation from the classical magnetoresistance at low *T* and *B* = 0 T due to weak antilocalization. The red line represents Equation (3) with dephasing exponent *p* = 1. (**c**) Temperature dependence of phase coherence length *l_ϕ_* plotted on a logarithmic scale. The red solid line shows the prediction of the 2-dimensional carrier model, which gives rise to the power-law dependence with exponent of −1/2.

The theoretical framework based on the two-carriers model, expressed in Equations (1) and (2), however, does not include quantum interference effects such as the weak antilocalization (WAL), which appears as additional features in *ρ_xx_*(*B*) curves around *B* = 0 at low temperatures. In order to extract these effects, the fit obtained by Equation (1) was subtracted from the experimental curves and the results are shown in Figure 3a as open circles. The obtained dip structures at low magnetic fields can be satisfactorily simulated within the framework of the WAL model constructed by Hikami et al. [39]. Figure 3b displays the zero field WAL strength as a function of *T*. The WAL correction to the classical *T* dependence of *ρ_xx_* for a diffusive, 2-dimensional system follows the relation [40]:

(3) $$∆\rho_{xx}\left(B=0,T\right)=\frac{e^{2}\tilde{p}\rho^{2}}{4\pi^{2}\hbar t}\ln\left(\frac{T}{T_{0}}\right),$$

where *T*_0_ is the characteristic temperature for the onset of decoherence, *t* is the film thickness, *ρ* is the resistivity, *e* is the elementary charge, and $\tilde{p}$ is the exponent in the quantum dephasing power law for the phase coherence length *l_ϕ_* ~ $T^{-\tilde{p}/2}$. Fitting Equation (3) to the data yields the red line in Figure 3b. From this fit we obtained the power law dephasing exponent $\tilde{p}$ ≈ 1.05 ± 0.07, which is expected for a 2-dimensional electron system [3], while the obtained characteristic temperature is *T*_0_ ≈ 30 K ± 4 K. Following the Hikami, Larkin, Nagaoka (HLN) theory [39], the correction to the resistivity Δ*ρ_xx_*(*B*), due to the weak antilocalization in the 2-dimensional case, can be described as [13,22,41]:

(4) $$∆\rho_{xx}\left(B\right)=\frac{e^{2}\rho^{2}}{2\pi^{2}\hbar t}\left[\Psi \left(\frac{B_{0}+B_{so}}{B}\right)-\frac{3}{2}\Psi \left(\frac{B_{\phi }}{B}+\frac{4B_{so}}{3B}\right)+\frac{1}{2}\Psi \left(\frac{B_{\phi }}{B}\right)\right]$$

where Ψ(x) is the digamma function, and

(5) $$B_{0,so,\phi }=\frac{\hbar }{4el_{0,so,\phi }^{2}}$$

In the above formulas, *l_so_* is the spin-orbit scattering length, *l_ϕ_* is the phase coherence length, closely related to the inelastic scattering length, while *l*_0_ is the elastic scattering length. Contribution from spin-spin scattering was neglected. During fitting, we constrained a scattering length *l*_0_ to the range of 15–100 nm according to other studies on Bi thin films [22].

The theoretical curves fitted to experimental points are shown in Figure 3a as solid lines, while the values derived from the fits are given in Table 1. As shown in Figure 3c the phase coherence length *l_ϕ_* increases from 30 nm to 90 nm as *T* is reduced from 20 K to 2 K. Similar values of *l_ϕ_* were obtained in [14] for arrays of Bi nanowires. Phase coherence length *l_ϕ_* indicated by the solid line in Figure 3c is proportional to *T*^−1/2^, which is expected for a 2-dimensional charge carrier system [42,43]. It should be noted that the system follows 2-dimensional behavior only if *l_ϕ_* > *t*. Indeed, for temperatures below 8 K *l_ϕ_* shown in Figure 3c is larger than the film thickness. For measurements carried out at temperature of 10 K and 20 K *l_ϕ_* ≤ *t* and the dimensionality of the sample for phase-coherent processes changes from two to three dimensions. This determines the limit of applicability of Equation (4), which for temperatures of 10 K and above can provide only approximate values of the scattering parameters.

Values of *l_so_* derived from analyzed data are approximately temperature independent and are in the range of 25–40 nm, showing a slight increase as temperature increases. The values of spin-orbit scattering length *l_so_* correspond to those obtained by others for polycrystalline Bi films. For example, Rabin et al. [22] derived *l_so_* in the range of 20–30 nm for films with thickness of 35 nm, while Hackens et al. [13] reported *l_so_* ≈ 100 nm for thicker Bi films of 80 nm. Our results fit between these reports, and for the analyzed temperature range are always smaller than film thickness. This confirms large spin–orbit interaction in thermally evaporated polycrystalline Bi thin films.

### 3.2. Antidot Arrays

**Figure 4:**
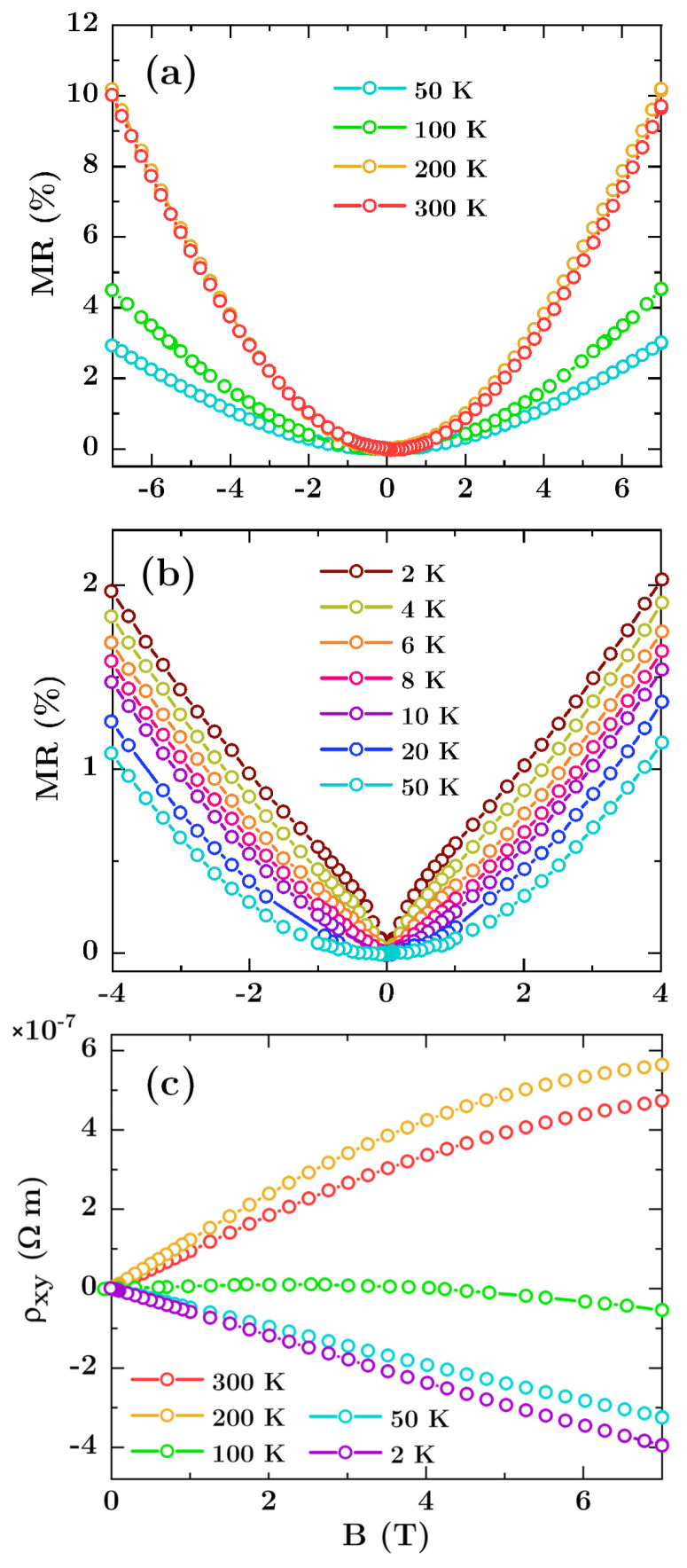
(**a**,**b**) Longitudinal magnetoresistance (MR) and (**c**) Hall transverse magnetoresistance vs field curves for Bi antidot arrays with antidot size of 65 nm. The results of the Hall measurements for temperature range 4 K–20 K were omitted for clarity, since they are similar to those obtained at 2 K and 50 K.

In order to study the influence of mesoscopic defects on the magnetotransport properties of Bi thin films, the measurements described for non-patterned layer have been performed for three hexagonally ordered arrays of Bi antidots with various diameters. The field dependence of longitudinal magnetoresistance MR(*B*) and transverse magnetoresistivity *ρ_xy_*(*B*) is shown in Figure 4. For the sake of brevity, we only present the results for antidots with diameter of 65 nm. The MR(*B*) and *ρ_xy_*(*B*) curves for two others arrays with antidot size of 45 nm and 110 nm are qualitatively similar and we present them in Figures S2 and S3 in the Supplementary Material.

**Figure 5:**
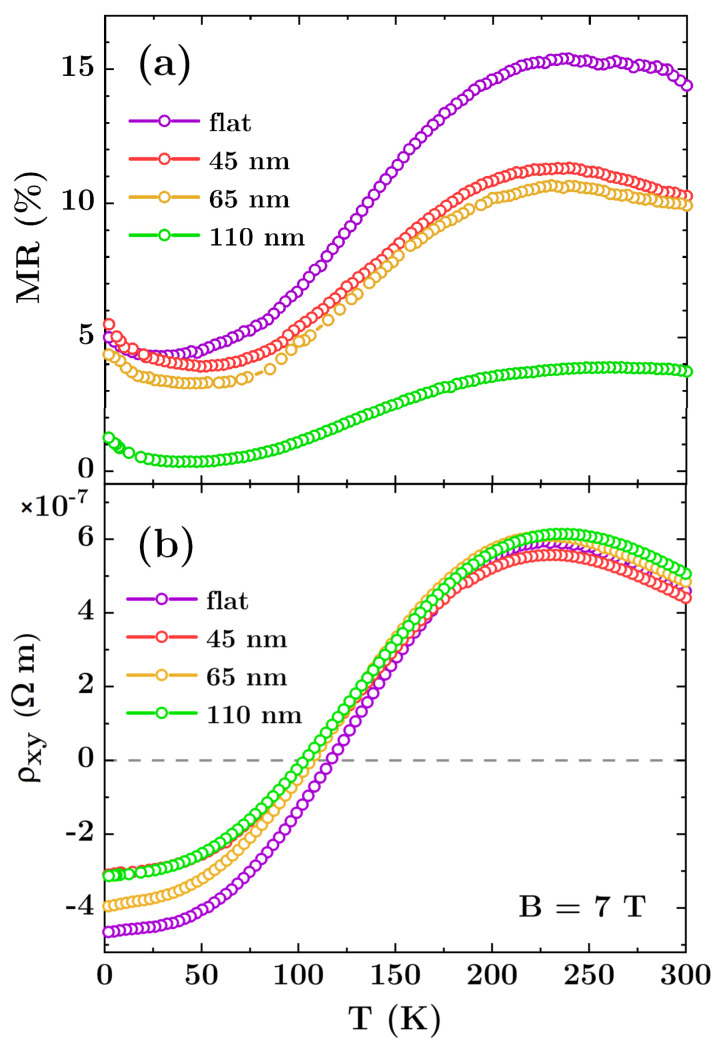
(**a**) MR values and (**b**) transverse resistance for flat reference film as well as for Bi antidot arrays with varying hole diameters. The values were measured at 7 T.

In comparison with the flat films, the introduction of holes increases the longitudinal resistivity *ρ_xx_* (see Table 1) up to 100% for antidots with diameter of 110 nm at 300 K. This effect is directly related to the change of geometry and the reduction of the effective cross-section of the film along current flow. Additionally, the introduction of antidots increases the density of the scattering centers, and reduces the mean free path of the charge carriers, which results in a resistivity rise. Similarly to the flat layers, the MR(*B*) curves show B-quadratic behavior without saturation at temperature above or equal to 20 K, which indicates the classical MR. As shown in Figure 5a, the largest MR values occurs in the temperature range of 200–250 K, however the maximum values for the arrays are smaller than those for the reference sample, and are 11.5%, 10.5% and 4% for antidots with diameter of 45 nm, 65 nm, and 110 nm, respectively. The degradation of MR for the larger antidots suggests that the edge effects at the nanostructure boundaries play important role in scattering of charge carriers, and thus modify the magnetotransport characteristics. It is well known that impurities, disorder and additional phases emerging at antidot edges can significantly change magnetic and electronic properties of the percolated thin films [44,45,46,47,48], and similar mechanism is expected in the studied Bi antidot arrays. On the other hand, Bergman, Tornow et al. [23,49,50] showed that magnetoresistance changes can be also understood by considering current distortions which appear around inclusions or voids in the host material. Such distortions become larger for stronger magnetic field and eventually may interact with each other or with neighbouring antidots. In special cases the interference between current distortions is destructive, which results in a decrease in magnetoresistance.

The evolution of the transverse resistivity with temperature, see Figure 5b, and change of the sign of the Hall coefficient clearly show a transition from p-type to n-type conduction with increasing temperature similarly as for reference the flat film. This suggests the patterning does not affect the character of bismuth conductivity. However, in the case of antidot arrays the procedure of fitting within the framework of the two-carrier model cannot be applied since the changes in resistivity of these systems have their source in geometrical factors and are not the result of the changes in the material transport parameters. This is not taken into account by Equations (1) and (2) therefore, fitting procedure applied to the data for antidot arrays would give unphysical results. However, considering the same preparation conditions and the same character of MR(B) and *ρ_xy_*(*B*) curves, especially the same temperature for which *ρ_xy_*(*T*) ≈ 0, we can assume that the carriers concentrations and mobilities are similar for all the samples.

**Figure 6:**
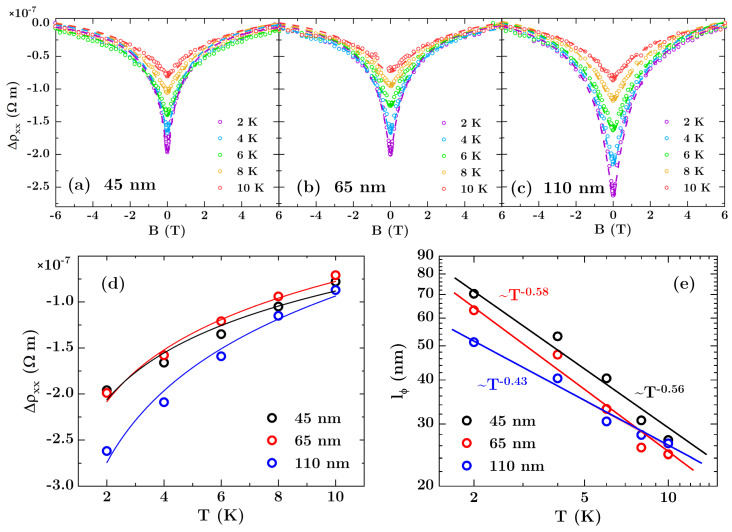
(**a**–**c**) Longitudinal magnetoresistivity for Bi antidot arrays after subtracting the B-quadratic background. Dashed lines represent theoretical curves fitted to experimental data using Equation (4) and Hikami-Larkin-Nagaoka (HLN) theory. The mean hole diameter is (**a**) 45 nm, (**b**) 65 nm, and (**c**) 110 nm. (**d**) Resistivity deviation from the classical magnetoresistance at low *T* and *B* = 0 T due to weak antilocalization. The solid lines represent fit with Equation (3). (**e**) Temperature dependence of phase coherence length *l_ϕ_* plotted on a logartithmic scale. The solid lines show the curves obtained by fitting the data to a power-law function of temperature *l_ϕ_* ~ *T^−x^*.

The MR data collected for all the arrays indicate the presence of WAL at low *T* and low *B*, in the form of a dip in the resistivity near *B* = 0. The longitudinal resistivity curves *ρ_xx_*(*B*) after subtracting the B-quadratic background are shown in Figure 6a–c as point series. As *T* or *B* are increased, the classical MR of bismuth dominates the data, similarly to observations from Rabin et al. for Bi films deposited on patterned silicon and AAO [22]. In all cases similar WAL-related MR of about 0.5% was recorded, but the largest absolute value of WAL strength of −2.62 × 10^−7^ Ωm was observed for antidots with a size of 110 nm. The *T* dependence of WAL correction to the *ρ_xx_* is presented in Figure 6d together with fits according to Equation (3). The increase of the absolute values of WAL-related MR signal for the arrays originates directly from an increase in the resistance of the films caused by higher defect density emerging from edge of the antidots—the larger the hole, the bigger its circumference and the higher defect density.

Following the HLN model, the WAL corrections were analyzed by fitting Equation (4) to experimental data as depicted in Figure 6a–c by dashed lines. The obtained curves reproduce the experimental points well, validating that Equation (4), originally introduced to formulate the WAL effect in 2D electron systems, can be also used for patterned systems [3]. Values derived from the fits are given in Table 1. Spin-orbit coupling lengths *l_so_* are approximately temperature independent, always smaller than film thickness, and comparable to those for flat film, which signifies strong spin-orbit coupling in Bi antidot arrays. The similar *l_so_* values were reported in [21,22] for Bi antidots fabricated on AAO.

Obtained *l_ϕ_* values plotted as a function of T are presented in Figure 6e showing exponential decline with temperature. It can be seen that the bigger the antidot size, the smaller *l_ϕ_* values at low *T*, which is associated with the higher probability of scattering events. The power law dephasing exponent $\tilde{p}$ values were extracted using *l_ϕ_*(*T*) data. For antidots with diameters of 45 nm and 65 nm, they agree within the measurement uncertainty with the value for the reference film. The exceptions are the biggest antidots, where $\tilde{p}$ is significantly lower than 1. This may indicate that carrier transport in this array has an intermediate dimensionality between 1D and 2D, since one should expect $\tilde{p}$ = 2/3, 1, and 3/2 for 1D, 2D, and 3D electron systems, respectively [3]. The 1D–2D crossover are observed usually in nanowires when *l_ϕ_* approaches cross section dimensions of the wire. Here, for antidots with diameter of 110 nm and period of 175 nm, the neck width between two neighboring holes reaches 65 nm and is close to *l_ϕ_* (*T* = 2K) = 51 nm. Therefore, the geometrical effect is plausible and could explain observed decrease of $\tilde{p}$. However, the effect was observed only for one size of antidots and in order to exclude the possibility of statistical variation of $\tilde{p}$ and to confirm the supposition concerning 1D-2D crossover, measurements for antidots bigger than 110 nm are necessary.

Despite the high order of antidots, no oscillations in MR(*B*) curves resulting from commensurability resonance were observed. Such resonances in the longitudinal magnetoresistance have been reported for semiconductor antidots, and usually are interpreted in terms of classical cyclotron orbits that are commensurate with the antidot period and symmetry [44,51,52]. The occurrence of such oscillations requires, however, the elastic mean free-path exceeding the array period, which is not the case of studied Bi arrays. As shown in Table 1, elastic scattering lengths *l*_0_ are always smaller than 100 nm and stay in the range of 30–40% of the period.

## 4. Conclusions

In summary, we investigated the influence of large area patterning, developed topography, and mesoscopic defects on the magnetotransport properties of Bi antidot arrays fabricated by nanosphere lithography and compared them to the flat reference layers. The simultaneous measurement of the transverse and the longitudinal magnetoresistance provided comprehensive information on transport properties and allowed us to obtain charge carriers density values and their mobilities as a function of temperature. The low-temperature MR showed a WAL effect from which *l_so_* spin-orbit scattering lengths, *l*_0_ elastic scattering lengths, and *l_ϕ_* phase coherence lengths were derived by assuming the HLN model. We showed that, in the absence of antidots, the charge carrier transport follows 2-dimensional behavior and the dimensionality for phase-coherent processes changes from two to three dimensions at temperatures higher than 10 K. For the biggest size of antidots, however, a decrease of the power law dephasing exponent $\tilde{p}$ was observed, which could indicate 1D–2D crossover caused by the geometry of the system. Such a 1D–2D crossover has not yet been reported for Bi antidots, which raises questions concerning its origin and potential use in sensorics. Our studies showed that the transport properties of Bi can be tailored by NSL complemented by the RF-plasma etching, leading to large area arrays with variable transport properties.

## Figures and Tables

**Table 1:** Summary of the parameters extracted from fits to WAL peaks with Equation (4) for flat reference Bi film and antidot arrays.

	Flat Film	Antidot Arrays		
		45 nm	65 nm	110 nm
*ρ_xx_* (*T* = 300 K, *B* = 0) (10^−6^ Ωm)	8.4	9.6	10.7	17
Δ*ρ_xx_* (*T* = 2 K, *B* = 0) (10^−7^ Ωm)	−1.63	−1.96	−1.99	−2.62
*l*_0_ (nm)	60–90	50–70	50–70	50–70
*l_so_* (nm)	25–40	20–30	25–30	25–30
*l_ϕ_* (*T* = 2 K) (nm)	86	71	64	51
$\tilde{p}$	1.05 ± 0.07	1.12 ± 0.14	1.16 ± 0.14	0.86 ± 0.08

## Supplementary Materials

The following are available online at https://www.mdpi.com/1996-1944/13/15/3246/s1, Figure S1: presents XRD pattern for 50 nm bismuth layer, Figures S2 and S3: present the field dependence of longitudinal magnetoresistance and transverse magnetoresistivity for antidots with diameter of 45 nm and 110 nm.
