# Supplementary material for: Weak Antilocalization Tailor-Made by System Topography in Large Scale Bismuth Antidot Arrays
Source: Materials (Basel). 2020 Jul 22;13(15):3246. doi: 10.3390/ma13153246 (PMC7436095; doi:10.3390/ma13153246)
Supplement: Supplementary file 1 [file materials-13-03246-s001.pdf]

materials

Article

Weak Antilocalization Tailor-Made by System Topography in Large Scale Bismuth Antidot Arrays

Michal Krupinski *, Arkadiusz Zarzycki, Yevhen Zabila and Marta Marszałek

Supplementary Material

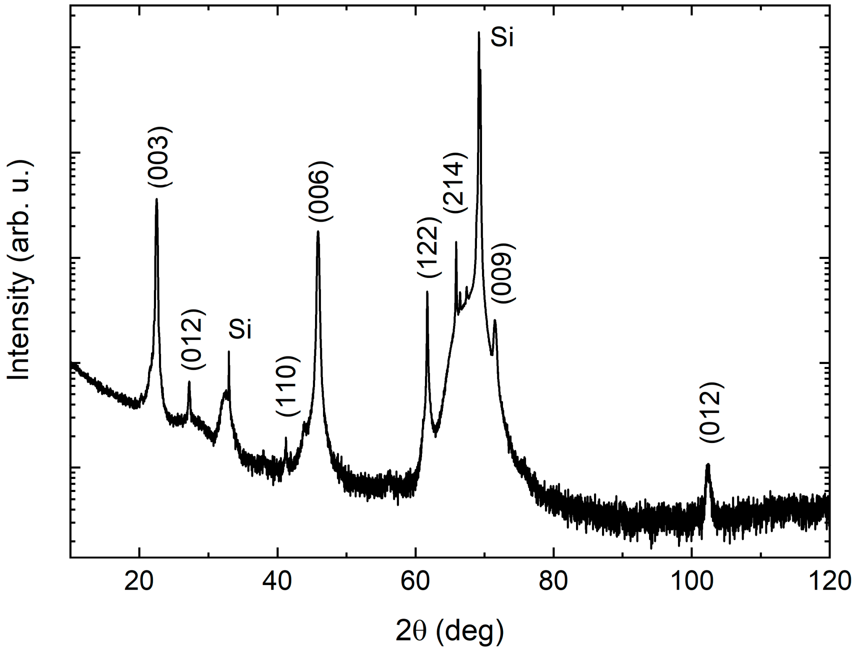

Figure S1. XRD pattern of the Bi (50 nm) thin film deposited on the flat Si/SiO_2 (100 nm) substrate.

Magnetotransport measurements for Bi arrays with antidot size of 45 nm and 110 nm.

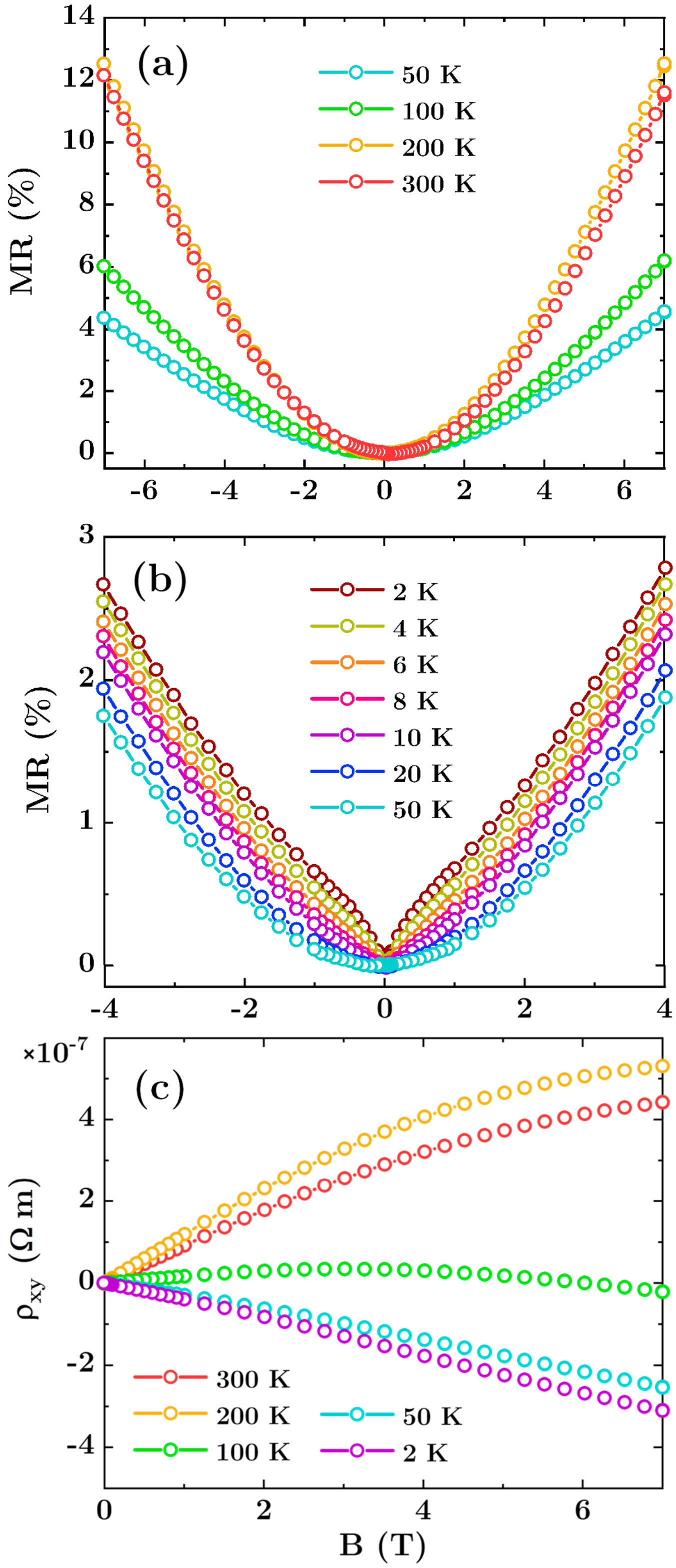

Figure S2. (**a**,**b**) Longitudinal magnetoresistance (MR) and (**c**) Hall transverse magnetoresistance vs field curves for Bi antidot arrays with antidot size of 45 nm. For clarity, the results of Hall measurements for temperature range 4 K – 20 K were omitted since they are similar to those obtained at 2 K and 50 K.

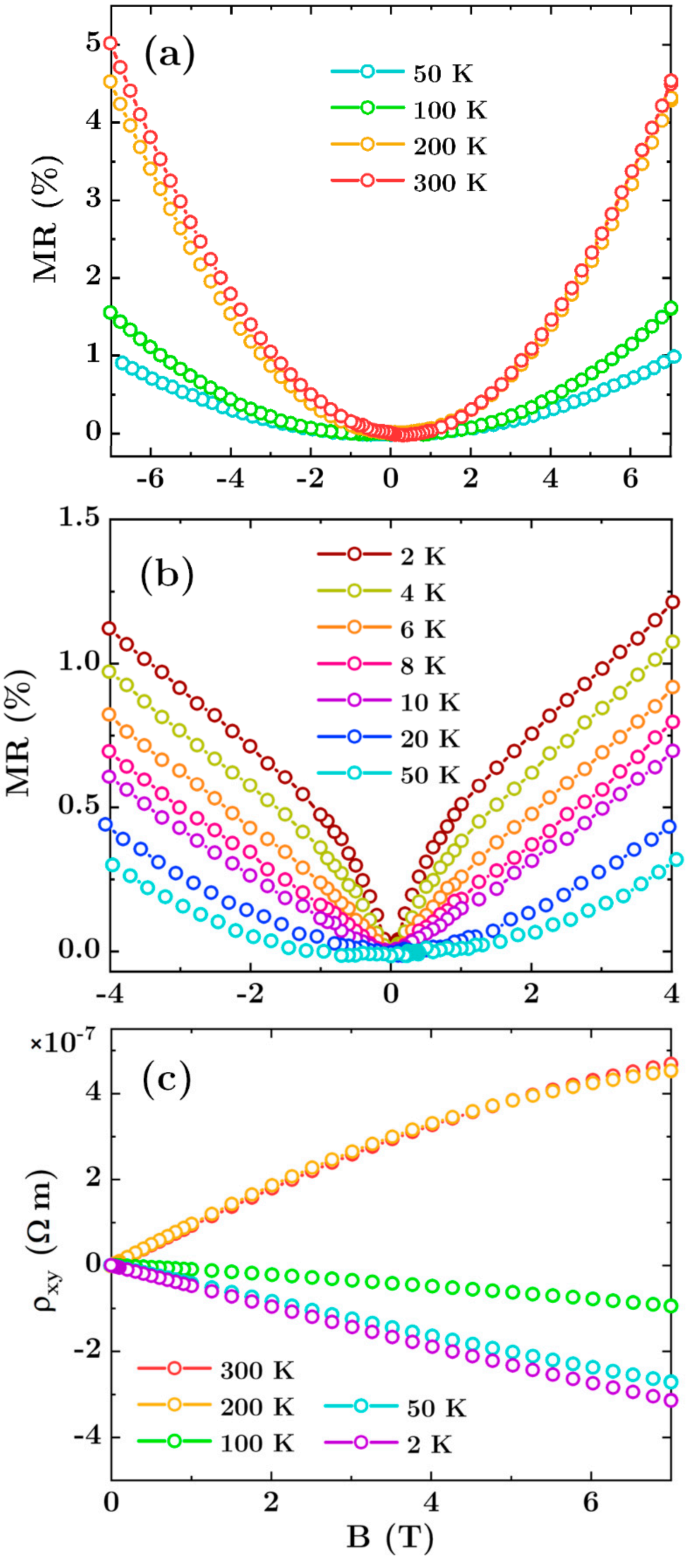

Figure S3. (**a**,**b**) Longitudinal magnetoresistance (MR) and (**c**) Hall transverse magnetoresistance vs field curves for Bi antidot arrays with antidot size of 110 nm. For clarity, the results of Hall measurements for temperature range 4 K–20 K were omitted since they are similar to those obtained at 2 K and 50 K.

© 2020 by the authors. Submitted for possible open access publication under the terms and conditions of the Creative Commons Attribution (CC BY) license (http://creativecommons.org/licenses/by/4.0/).
